# The imbalance in expression of angiogenic and anti-angiogenic factors as candidate predictive biomarker in preeclampsia

**Published:** 2015-05

**Authors:** Pooneh Nikuei, Kianoosh Malekzadeh, Minoo Rajaei, Azim Nejatizadeh, Nasrin Ghasemi

**Affiliations:** 1 *Molecular Medicine Research Center, Hormozgan University of Medical Sciences, Bandar Abbas, Iran.*; 2 *Fertility and Infertility Research Center, Hormozgan University of Medical Sciences, Bandar Abbas, Iran.*; 3 *Research and Clinical Center for Infertility, Shahid Sadoughi University of Medical Sciences, Yazd, Iran.*

**Keywords:** *Preeclampsia*, *Angiogenic proteins*, *Biomarker*, *Vascular endothelial growth factor* (*VEGF*-*A*), *Vascular endothelial growth factor receptor*-*1* (*VEGFR*-*1*)

## Abstract

Preeclampsia is an important pregnancy disorder with serious maternal and fetal complications which its etiology has not been completely understood yet. Early diagnosis and management of disease could reduce its potential side effects. The vascular endothelial growth factor (VEGF) family including VEGF-A is the most potent endothelial growth factor which induces angiogenesis and endothelial cell proliferation and has basic role in vasculogenesis. VEGF and its tyrosine kinase receptors (Flt1 and KDR) are major factors for fetal and placental angiogenic development. Finding mechanisms involved in expression of angiogenic factors may lead to new prognostic and therapeutic points in management of preeclampsia. Recent researches, has shown capability of some anti-angiogenic factors as potential candidate to be used as early predictors for preeclampsia. Soluble fms-like tyrosin kinase-1 (sFlt1) is a truncated splice variant of the membrane-bound VEGF receptor Flt1, that is produced by the placenta and it can bind to angiogenic growth factors and neutraliz, their effects. It is also observed that the ratio of sFlt1 to placental growth factor is valuable as prognostic marker. In this review, VEGF family member’s role in angiogenesis is evaluated as biomarkers to be used for prediction of preeclampsia.

## Introduction

Preeclampsia (PE) is a life threatening complication of pregnancy which is diagnosed by hypertension and proteinuria after 20 weeks of gestation. PE is a multiple organ syndrome that affects at least 5% of all pregnancies worldwide (1,2). About 75000 mothers and 500000 neonates die every year around the world because of the PE complications (3). It is a major cause of maternal and fetal mortality and morbidity (4). Early diagnosis and treatment of disease can reduce its potential fetal and maternal side effects. Because of unknown pathophysiology, it is referred as the disease of theories (5). Women with PE are more prone to cardiovascular disorders later in their life (6). According to the onset time, the disease is classified to early (before 34 weeks) and late onset (after 34 weeks) (7). There is difference in the origin of PE regarding to onset time. While the cause of early onset PE is most related to inadequate placentation and angiogenesis balance, the late onset PE is associated with long term cardiovascular risk factors like obesity, diabetes and hypertension (8).

PE could also result in HELLP syndrome (hemolysis, elevated liver enzymes, and low platelets) and seizures (eclampsia) (9). Low birth weight, prematurity and death are its fetal complications (10). Placenta removal could improve the symptoms but it threats neonatal health due to serious complications such as early preterm delivery and prematurity (11). Conditions that increase the risk of PE are chronic hypertension, diabetes mellitus, renal disease, obesity, hypercoagulable states such as antiphospholipid syndrome and factor V Leiden, advanced maternal age and conditions associated with increased placental mass such as multifetal gestations and hydatidiform mole (12). Angiogenic factors imbalance in preeclampsia result in decreased activity of proangiogenic factors in association with high activity and expression of the anti angiogenic factors (13). Among angiogenic factors, vascular endothelial growth factor (VEGF) and its receptors look to play a major role in not only physiological but also pathological angiogenesis like cancer (14).

**Angiogenesis in normal pregnancy and preeclampsia**

Adequate nutrient and substrate supply are necessary for placental and fetal normal intrauterine development. Uterine blood supply disorders are associated with higher risk for preterm delivery, PE and intrauterine growth restriction (15). The placenta is a significant organ for fetal development. Not only fetus exchange of gases, nutrients, and waste products perform through placenta but also it protects fetus from rejection by maternal immune system. During fetal development high levels of angiogenesis and vasculogenesis take place in placenta (16).

A number of causes for placental dysfunction are hypoxia, oxidative stress, reactive oxygen species, catechol-O-methyltransferase deficiency, hemoxygenase deficiency, and immunologic/inflammatory factors that result in imbalance between angiogenic and anti-angiogenic factors (17). Vasculogenesis, angiogenesis and pseudovasculogenesis are three stages of placental vascular network development. Vasculogenesis that takes places in first weeks of gestation is stage in which transformation of a subpopulation mesenchymal precursor cells into hemiangioblastic endothelial precursors take places. New blood vessels formation in the placenta is due to differentiation of these cells (18). Vasculogenesis is followed by angiogenesis, which begins at day 21 of pregnancy. Angiogenesis is formation of new vessels from pre-existing vessels. Soluble angiogenic factors expressed in the trophoblasts of the placenta, maternal decidua and macrophages stimulate capillary formation in the chorionic villi of the placenta. Development of capillary beds of the villi will continue until week 26 of gestation. From 26 weeks of gestation to delivery time villous vascular growth will mainly done by non-branching angiogenesis due to the formation of mature intermediate villi that contain poorly branched capillary loops (19). VEGF is a crucial inducer for branching angiogenesis (20). Pseudovasculogenesis or epithelial-endothelial transformation is a key event during vascular development of placenta. The result of this stage is remodeling of spiral arteries from high resistance and low flow muscular vessels to sac like vessels with low resistance and high flow which leads to the increased blood flow to fetus (19, 21).

Insufficient maternal spiral arterial remodeling, disability of cytotrophoblast cells to obtain endothelial-like phenotype and invasion defect to myometrial spiral arteries result in narrow myometrial segments (22-24). This pathologic process leads to release of series of pro-inflammatory factors from hypoxic placenta and following that damage of maternal circulatory system happens. Therefore PE has two stages, first is poor placentation in early gestation and second stage is maternal system dysfunction (25). There is evidence that show VEGF and transforming growth factor-β1 (TGF-β1) are necessary for endothelial health and normal pregnancy and normal vascular homeostasis (19). Placental ischemia causes higher production of anti-angiogenic proteins like soluble fms-like tyrosin kinase-1 (sFlt1) and soluble endoglin/CD105 (sEng) that have serious effects on cardiovascular system by disrupting normal placental angiogenesis. Release of these placental anti-angiogenic factors into maternal circulation lead to damage of maternal endothelium and clinical manifestations of preeclampsia such as hypertension and PE (12). Genetic explanations are suggested for the overproduction of anti-angiogenic factors in preeclampsia. Understanding mechanisms involved in disrupting angiogenesis in pregnancy may provide novel preventive and therapeutic points in early management of PE.

**VEGF**

The VEGF family including VEGF-A, VEGF-B, VEGF-C, VEGF-D, VEGF-E, and placental growth factor (PlGF). VEGF-A is one of the most potent endothelial growth factors. It induces angiogenesis and endothelial cell proliferation and has basic role in vasculogenesis. Splice variants of VEGF-A including 121, 165, 189, and 206 amino acids, each has a specific exon addition. Among these variants the most predominant protein is VEGF 165 (26, 27). VEGF and its tyrosine kinase receptors (VEGFR-1/Flt1 also called fms-like tyrosine kinase-1 and VEGFR-2/KDR/Flk-1) are major factors for fetal and placental angiogenic development. Biological VEGF activity is mediated through interaction with its receptors Flt1 , that is expressed in vascular endothelial cells in placental trophoblast cells in macrophages and in monocytes and VEGFR-2 (KDR1/Flk1), a potent receptor tyrosine kinase that is primarily expressed in vascular endothelial cells especially during vascular development (28, 29).

The kinase activity of VEGFR-1 is relatively weak compared with that of VEGFR-2. VEGF is highly expressed in the early human placenta. Immunohistochemical and in situ hybridization studies showed that villous trophoblast and Hofbauer cells are the main source of this cytokine (30, 31). VEGF promotes nitric oxide and vasodilatory prostacyclins in endothelial cells, and plays its role by decreasing vascular tone and blood pressure (32, 33). Expression of *VEGF* gene is induced by hypoxia as a strong exciter and it enhance VEGF mRNA stability (34, 35). Under in vitro conditions, VEGFA induces cytotrophoblast invasion, which can be blocked by addition of sFlt1 (36). In cancer patients received VEGF inhibitor drugs PE symptoms like hypertension, proteinuria and glomerular damage was observed (37, 38). Also there is evidence indicating that women affected to PE have decreased long-term risk for malignancy (39).

**PlGF**

In VEGF family, PIGF is closely related to VEGF-A and is another pro-angiogenic factor secreted by placenta. PlGF is expressed in syncytiotrophoblast layer of the placenta with direct contact with maternal circulation (40) and have several different isoforms (PlGF-1, -2, -3, and -4) (41). PIGF induce its role in angiogenesis by binding to Flt1 (42), unlike VEGF-A which binds to both Flt1 and KDR, PlGF could not bind to KDR (43, 44). PIGF may raise angiogenesis by replacing VEGF from Flt1 and shift VEGF towards KDR with kinase activity about ten-fold higher than that of Flt1 (25, 45).

PlGF and VEGF could also bind to soluble and splice variant form of Flt1 (sFlt1) and be deterred from acting with their main receptors (16). PlGF concentrations increase during normal pregnancy significantly at 28-32 weeks but Levels of free VEGF decreases with progression of pregnancy (46). Most studies show that the level of free PlGF in maternal blood decreases in PE. This reduction could help in early diagnosis of PE because it occurs few weeks before clinical presentation of disease. In early onset PE, maternal serum PlGF levels in weeks 21-32 of gestation are lower compared with late onset PE, in severe forms compared with mild forms and in association with small-for-gestational age compared with isolated PE (46, 47).

**VEGF and PlGF expression in preeclampsia**

Most of serological studies about circulating angiogenic factors in PE reported decreased circulating levels of free VEGF and PlGF, which has been associated with increased circulating sFlt1, a splice variant of the VEGF receptor Flt1 (46, 48, 49). In study of Levin *et al *observed that women who later affected to PE had lower PlGF levels in weeks 13-16 pregnancy with the highest difference in concurrent with sFlt1 rising (46). Helske *et al* study showed that PlGF concentrations in maternal plasma were lower in preeclamptic patients (50). But Savvidou *et al* reported that PE could not be preceded by alteration in urinary PlGF concentration in first trimester of pregnancy (51).

Cooper *et al* found that levels of VEGF mRNA were significantly lower in the preeclamptic women compared with the control women (52). Polliotti *et al* studied on severe, early onset preeclamptic patients and reported that PlGF and VEGF were significantly lower in patients than in controls (53). Kim* et al* also reported decreased expressions of VEGF in both level of mRNA and protein in placenta of preeclamptic patients compared with the normotensive controls (54). Andraweera *et al* reported mRNA placental expression of VEGFA and PlGF were reduced in preeclamptic patients compared to normal control pregnancy (55). In contrast, three studies showed the increase in expression of angiogenic factors such as VEGF in preeclampsia (26- 57). This contrast is also observed in microarray studies. In Lee *et al* study with aim of investigating cytokine-and oxidation-related genes or preeclampsia using DNA microarray analysis they found up-regulation of VEGFA mRNA that were confirmed using quantitative real time-polymerase chain reaction (QRT-PCR) (56). But Jarvenpaa *et al* showed down-regulation of VEGF in both early and late onset PE by microarray (58). Ranheim *et al* reported that there were no statistically significant differences in expression of VEGF in mRNA levels between the preeclampsia and the control group for either the decidual or placental tissues (59). Sgambati *et al* reported that in the cases of preeclampsia, the levels of VEGF mRNA were the same as the control group (60).

These findings consider that circulating angiogenic proteins have a crucial role in PE pathogenesis. However, it seems that VEGF and PlGF are two most studied serum markers for PE. According to most studies their free levels decreased in PE accompanied with increase in serum level of sFlt1. In PE excess sFlt1 binds to VEGF and PlGF and antagonizes these proangiogenic molecules resulting in preventing them to interact with their main cell surface receptors Flt1 and KDR and cause endothelial dysfunction. Despite significant role of VEGF-A in PE pathogenesis, it seems that maternal VEGF-A levels have a limited role as PE predictor, unlike PlGF with capability as a biomarker in preceding PE (61).

**Flt1 and sFlt1 status in preeclampsia**

Flt1 is detected in the primitive vascular lumens and angiogenic cell cords (62). *Flt1 *gene produces two mRNAs in placenta and vascular endothelial cells, a long form for the full-length receptor Flt1 and a short form for sFlt1 which carries only the ligand binding region (14). Alterations in these two forms are reported in studies related to PE. Most of studies except a few studies showed up-regulation of Flt1 mRNA in preeclampsia (55, 63). For instance, Chung *et al* study showed that Flt1 mRNA and protein both increase in preeclamptic pregnancies (57). Up-regulation of Flt1 was also reported by Nishizawa *et al* in severe preeclamptic pregnancies and unexplained fetal growth restriction (64). Munaut *et al* studies on 30 severe PE women compared to 30 normal pregnancies showed VEGFR-1 mRNA up-regulation and increased its plasma level in severe PE (65). Tripathi* et al* reported elevation of sFlt1 in serum and also up-regulation of Flt1 in placenta (66). Levine *et al* reported that the level of sFlt1 increase in placenta at 5^th^ week before onset of clinical symptoms of PE (46).

Serum levels of sFlt1 observed to be higher in earlier onset PE compared with late onset, in severe PE compared with mild disease and in association with small-for-gestational-age compared with isolated PE (46, 49, 67, 68). In microarray study done by Jarvenpaa *et al* up-regulation of Flt1 was also reported (58). 

In global placental gene expression profile by microarray in severe preeclampsia done by Sitras *et al*, up-regulation of Flt1 was deduced (69). Lee *et al *showed up-regulation of Flt1 by using microarray analysis. Their results were confirmed using quantitative real time-polymerase chain reaction (QRT-PCR) (56). Toft *et al* studied on the transcriptomes of placental tissues from PE and small for gestational age (SGA) pregnancies by whole-genome microarray and quantitative Real time PCR. The authors mentioned increased expression of Flt1 was detected by QRT-PCR in the PE‏‏+SGA group but microarray analysis did not reveal any significant differences between groups (70).

Bdolah *et al* reported that the rate of circulating sFlt1 to PlGF was significantly increased in women carrying fetus with trisomy 13 (71). Regarding to the position of *Flt1 *gene on chromosome 13 it could be resulted in increased risk of PE in the women carrying fetus with additional copy of chromosome 13. Foyouzi *et al* reported no significant difference in levels of sFlt1 and VEGF in cerebrospinal fluid (CSF) of patients with preeclampsia compared with normotensive controls (72). Meynard *et al* injected exogenous sFlt1 to pregnant rats. Consequently, the symptoms of preeclampsia such as hypertension, proteinuria and glomerular endotheliosis have been observed (49). This increase in Flt1 expression in PE could derived from dual role of Flt1 in angiogenesis, a negative role in early embryogenesis and a positive role in cancer and other diseases. Its negative role in early embryogenesis maybe resulted from its strong binding and neutralizing of VEGF via the ligand-binding domain (14).

In the other hand, increase in level of circulating sFlt1 is also observed in most studies related to PE (9, 46, 67, 73-79). Levels of sFlt1 rise at 33-36 weeks at pregnancy (46). The concentration of sFlt1 and PIGF had been measured in different studies summarized in table I. As it can be seen the serum levels of sFlt1 are increased in preeclampsia associated with decreased levels of free VEGF and PIGF. Alternative mRNA processing has major role to produce sFlt1 in such a way the transmembrane and cytoplasmic domains are eliminated (80). This molecule can potentially release into maternal circulation, binds and inhibits VEGF and PIGF (81). sFlt1 antagonizes pro-angiogenic proteins such as VEGF and PlGF, which are essential for normal vascular endothelial homeostasis(82).

Levine *et al* found that the sFlt1 concentrations in controls remained constant until 33-36 weeks of gestation but increased in women who later had PE beginning 11 to 9 weeks before the onset of PE. More rapid increase in the sFlt1 levels observed within five weeks before the onset of preeclampsia. In the other hand, the PlGF concentrations began to decrease 11 to 9 weeks before the onset of preeclampsia, with substantial reductions during the 5 weeks before the onset of hypertension or proteinuria (46).

Also sFlt1 circulating levels rise before disease onset and correlates with disease severity and is related with proteinuria and hypertension onset time inversely (36, 83). Levein *et al *had also reported that alterations in the sFlt1 and PlGF levels were more pronounced before the onset of PE in women who had preeclampsia before term (<37 weeks of gestation) than in women who had an onset of preeclampsia at term (37 weeks) (46). Verlohren *et al* reported that in healthy pregnant patients, median serum concentrations of sFlt1 increased continuously from a lowest serum concentration of 1445 pg/mL in 10-14 weeks of gestation to a highest serum concentration of 4400 pg/mL in >37 weeks of gestation. The PIGF serum concentrations in normal pregnancies showed a continuous increase until the middle of the third trimester and then a decrease to the end of pregnancy. Overall PIGF serum concentrations ranged from 25.89-2096 pg/mL. Median PIGF serum concentrations ranged from a minimum of 62.8 pg/mL in 10-14 weeks of gestation to a maximum of 439 pg/mL in 29-33 weeks of gestation (84). 

**KDR (FLK or VEGFR-2) and preeclampsia**

In some studies no difference in expression of KDR mRNA between PE and controls was observed (26, 50, 57, 65). However, down-regulation of placental KDR expression mentioned in some studies (55, 63, 85). Also low plasma concentrations of soluble KDR are reported by Chaiworapongsa *et al* in PE and small for gestational age (86). The soluble form of the KDR receptor is hypothesized to be generated due to alternative mRNA splicing or proteolysis cleavage of the membrane-bound receptor and also considered as an anti-angiogenic protein with unknown mechanism of action (61).

**Clinical sensitivity and specificity for sFlt1 / PIGF as candidate prediction biomarker**

It appears that PlGF deficiency and sFlt1 excess may result from placental hypoxia associated with incomplete remodeling of maternal spiral arteries. The incompletely remodeled arteries offer persistently high resistance to uterine artery blood flow, and they may be predisposed to vascular rupture in the placental bed, especially after the onset of hypertension (87). 

Numerous studies have consistently demonstrated elevated serum levels of sFlt1 and decreased free PlGF in women with preeclampsia compared with normal pregnancies (Table I). Most studies agreeing that the higher the sFlt1 level, the more predictive for PE especially for early onset of clinical disease. Importantly, this increase in serum sFlt1 levels is detectable up to 5 weeks before the onset of clinical symptoms. Altered concentrations of angiogenic and anti-angiogenic peptides observed not only in the maternal circulation in PE but also in placental implantation disorders such as IUGR, small-for-gestational-age (SGA) births, fetal death of unexplained etiology, and twin-to-twin transfusion syndrome (26, 46, 49, 88-96).

As discussed in above, reduction in serum level of PlGF in preeclamptic women was observed in different studies (46, 49, 68, 84, 97-104). It seems that decrease of free PlGF is because of elevation of sFlt1 levels, which bind to PlGF and consequently neutralize it. This reduction in serum PlGF is seen 9-11 weeks before the development of hypertension and proteinuria. Considerable reduce observed in the 5 weeks before the onset of disease (46, 98, 100, 105). sFlt1 was found to be generally higher in the pre-eclamptic group in both 2^nd^ and 3^rd^ trimester. Therefore its diagnostic accuracy, sensitivity, specificity were lower than PlGF, endoglin and sFlt1: PlGF ratio. This result is probably due to the late increase of sFlt1 in PE patients (1). It has been reported that sFlt1 levels are not different between preeclamptic patients and controls at 17 weeks of gestation and that this difference becomes significant only a few weeks before the onset of the clinical signs of the disease (115).

The sFlt1/PlGF ratio as an anti-angiogenic activity index shows alterations in both sFlt1 and PlGF and it is a better predictor of PE than either measure alone (83). sFlt1/PlGF ratio was found to be more strongly associated with PE because, as reported in literature, it reflects the balance between sFlt1 and PlGF that is modified in the preeclamptic group (91). However, it is difficult to measure free VEFG due to its low concentration in PE and PlGF levels and sFlt1/PlGF ratio during mid-gestation are better tools for prediction of PE (83). A recent study about clinical validity of sFlt1/PlGF showed because of low sensitivity of test it could not be used as a diagnostic test alone, but it could be one element beside other tests (116).

The calculated sFlt1 /PIGF ratio was summarized in table II. According to study of Verlohren* et al* lowest values were observed in gestational weeks 24-28 with a median sFlt1/PIGF ratio of 3.80. Higher values were detected in the beginning and end of pregnancy, with sFlt1 /PIGF ratio of 22.7 in weeks 10-14 and a sFlt1 /PIGF ratio of 26.2 in pregnancies more than 37weeks (84). In most of studies the sensitivity of more than 75% is demonstrated for prediction value of sFlt1 /PIGF ratio especially for early onset preeclampsia (Table II).

The diagnostic power of the sFlt1 /PlGF ratio appears to be greater in patients with early-onset preeclampsia compared with late onset (117). 

Stephan *et al* suggested the combination of uterine artery Doppler (UAD) with measurement of sFlt1 / PlGF ratio can predict early-onset preeclampsia with 83% sensitivity and 95% specificity (110). Lim *et al* deduced that more reliable prediction using the combined ratio of (sFlt1 +sEng)/ (PlGF+ TGF-β1) could expand the clinical window for prevention of PE (109).

**Methodology**

In order to collect papers on preeclampsia and angiogenic and anti-angiogenic factors, a comprehensive literature review was conducted in PubMed, Medline, Science Direct, Cochrane, and Google Scholar. We used the following keywords to retrieve related publications: "Pre-eclampsia” OR “Preeclamsia” OR “PE” AND "Aniogenesis” OR “Antiangiogenesis” OR “Anti-angiogenesis” OR “Angiogenic factors “ OR “VEGF” OR “PIGF” OR “VEGF receptor” OR “VEGF-R” OR “Flt1 ” OR “ sFlt1 ” OR “soluble fms-like tyrosin kinase-1” OR “KDR” OR “FLK” OR “VEGFR-2”. The keywords were search in all fields in a paper.

For instance in PubMed the following search strategy was used: "Preecalmpsia" [All Fields] AND "Angiogenic factors "[All Fields] OR “angiogenesis” [All fields] OR “sFlt1 1" [All Fields]). Totally 415 papers were found, 118 papers were selected on preeclampsia that 29 papers contained measurement of angiogenic and anti-angiogenic factors level in blood of women with preeclampsia in different weeks of pregnancy.

**Table I:** The serum levels of sFlt1 and PlGF in women with preeclampsia demonstrated in main studies

**Study by**	**Weeks of sampling**	**sFlt1 (pg/ml)**		**Fold of increase**	**PIGF (pg/ml)**		**Fold of decrease**
		**Preeclampsia**	**Normal**		**Preeclampsia**	**Normal**	
Erez et al (106)	6-15 (PE>37)	1488	1788	0.8	26.2	35.4	1.3
Erez et al (106)	6-15 (PE<37)	1308	1788	0.7	20.3	35.4	1.7
Kusanovic et al (107)	12	1426	1726	0.8	24	34	1.4
Levine et al (46)	13-16	-	-	-	90	134	1.5
ThadhanI et al (108)	1^st^ trimester	1048	973	1.0	23	63	2.7
Kim et al (104)	16-18	3861	3353	1.1	86	146	1.7
Lim et al (109)	14-21	4945	2788	1.7	100	175	1.7
Kusanovic et al (107)	22	1637	1612	1.0	214	330	1.5
Stepan et al (110)	21-22	1927	452	4.2	119	184	1.5
Cripsi et al (111)	24	1257	526	2.4	92	426	4.6
Erez et al (106)	20-25 (PE>37)	1532	1799.5	0.8	273.4	345	1.3
Erez et al (106)	20-25 (PE<37)	1946	1799.5	1.0	126.3	345	2.7
Chaiworapongsa et al (67)	26-41	5063	1375	3.6	-	-	-
Sunderji et al (112)	20-36	91514	2416	37.8	12	447	37.2
De Vivo et al (113)	2^nd^ trimester	20330	7169	2.8	200	961	4.8
Ohkuchi et al (114)	32	10471	3019	3.5	53	549	10.4
Polliotti et al (53)	<34	-	-	-	61.3	122.4	2.0
Tsatsaris et al (97)	30-38	2690	120	22.4	67	586	8.7
Shibata et al (68)	34-35	5221	1857	2.8	86	228	2.6
De Vivo et al (113)	3^rd^ trimester	44870	12560	3.6	91	852	9.4
Verlohren et al (84)	Onset of clinical disease	12981	2641	4.9	76	342	4.5
Levine et al (46)	Onset of clinical disease	4382	1643	2.7	-	-	-

sFlt1: soluble fms-like tyrosin kinase-1

PlGF: placental growth factor

**Table II:** The sensitivity and specificity of sFlt1 /PlGF ratio test for prediction of PE mentioned in key studies

**Study by**	**PE** ** type**	**Cut off**	**Sensitivity**	**Specificity**
Kim et al (104)		1.40	80.4	78.0
Stepan et al (110)	PE + IUGR Early onset PE	3.15 4.67	63 71	50 76
Verlohren et al (84)	Early onset PE Late onset PE	85 85	89 74	97 89
De Vivo et al (113)		38.5	88.5	88.5
Ohkuchi et al (114)	Early onset PE	45	100	95
Sunderji et al (112)		137	96	97
Diab et al (118)	Early onset PE	7.77	100	90

sFlt1: soluble fms-like tyrosin kinase-1

PlGF: placental growth factor

PE: Preeclampsia

IUGR: intrauterine growth retardation

## Conclusion

PE is a pathologic complication of pregnancy that is closely related to placental dysfunction. In this disorder shallow invasion of cytotrophoblasts due to abnormal placentation will result in release of harmful pro-inflammatory molecules from placenta and end to maternal systemic dysfunction. Preeclampsia can be a potentially life threatening condition especially in developing countries and also it has been implicated in an increased risk of cardiovascular disease in later life (6). Genetic factors may role in pathogenesis of preeclampsia but exact mechanisms are not known yet. Different studies demonstrate the ability of cytokine analysis to differentiate preeclampsia from normal pregnancies. Studying about *Flt1 *gene regulation and its splicing may help for better understanding of preeclampsia pathogenesis and lead to prevention and treatment of the disease. It seems that the increased expression of Flt1 could disturb VEGF-mediated function on trophoblast and endothelial cells in preeclampsia (14).

Binding of free VEGF and PIGF with sFlt1 cause inhibition of interacting these angiogenic molecules with their main receptors. In PE, additional production of Flt1 and sFlt1 which cause endothelial dysfunction and anti-angiogenic state are likely related to placental hypoxia. Most of the circulating VEGF and PlGF are bound to sFlt1 which is produced higher than normal pregnancies and inhibits VEGF induced vasodilation effects. Inconsistence in studies about VEGF signaling in PE could be due to factors such as differences in geographic location of the studied population, different sample size and sampling methods, mode of delivery, and different technique methods. Large studies in different ethnicity in base of VEGF signaling may lead to find predictive marker as well as safe treatment for PE. Early prediction of PE could have great benefits for prenatal care and early treatment, so attention has been turned toward finding definite non-invasive test. Among biomarkers under investigation, angiogenic biomarkers like sFlt1, PlGF and sFlt1 to PlGF ratio are at the most advanced stage.

These angiogenic factors correlate with disease severity, could be detected several weeks before clinical presentation of the disease and have predictive value for diagnosis of severe-early onset PE but have a limited capacity in prediction of late onset PE and could not be used alone for intervention, but in combination with other angiogenic factors like soluble endoglin, Doppler sonography and other clinical and biochemical biomarkers they are more useful for predicting severe early onset PE.

## Conflict of interest

None of the authors have any conflict of interest.
